# Targeting Bruton’s tyrosine kinase (BTK) as a signaling pathway in immune-mediated diseases: from molecular mechanisms to leading treatments

**DOI:** 10.1186/s42358-024-00401-y

**Published:** 2024-08-21

**Authors:** Gita Manzari Tavakoli, Niloufar Yazdanpanah, Nima Rezaei

**Affiliations:** 1 Student’s Scientific Research Center, School of Medicine, Tehran University of Medical Sciences, Tehran, Iran; 2 Research Center for Immunodeficiencies, Children’s Medical Center, Tehran University of Medical Sciences, Tehran, Iran; 3 Network of Immunity in Infection, Malignancy and Autoimmunity (NIIMA), Universal Scientific Education and Research Network (USERN), Tehran, Iran; 4 Department of Immunology, School of Medicine, Tehran University of Medical Sciences, Tehran, Iran

**Keywords:** Bruton's tyrosine kinase, BTK, Bruton's tyrosine kinase inhibitor, BTKi, Immune-mediated diseases, Autoimmune diseases, Inflammatory diseases

## Abstract

Bruton’s tyrosine kinase (BTK), a nonreceptor tyrosine kinase, plays a remarkable role in the transmission and amplification of extracellular signals to intracellular signaling pathways. Various types of cells use the BTK pathway to communicate, including hematopoietic cells particularly B cells and T cells. The BTK pathway plays a role in controlling the proliferation, survival, and functions of B cells as well as other myeloid cells. First, second, and third-generation BTK inhibitors are currently being evaluated for the treatment of immune-mediated diseases in addition to B cell malignancies. In this article, the available evidence on the action mechanisms of BTK inhibitors is reviewed. Then, the most recent data obtained from preclinical studies and ongoing clinical trials for the treatment of autoimmune diseases, such as pemphigus vulgaris, pemphigus foliaceus, bullous pemphigoid, systemic lupus erythematosus, Sjögren’s disease, rheumatoid arthritis, systemic sclerosis, multiple sclerosis, myasthenia gravis, and inflammatory diseases such as psoriasis, chronic spontaneous urticaria, atopic dermatitis, and asthma are discussed. In addition, adverse effects and complications associated with BTK inhibitors as well as factors predisposing patients to BTK inhibitors complications are discussed.

## Introduction

In 1952, Ogden Bruton first reported X-linked agammaglobulinemia (XLA), which is a primary immunodeficiency disease, in an 8-year-old boy who complained of recurrent bacterial sepsis, otitis, and osteomyelitis, manifested by a notably decreased B-cell number and decreased serum immunoglobulin levels [1]. In 1993, the genetic basis of XLA was discovered as a mutation in a coding sequence of protein-tyrosine kinase and named after Bruton as Bruton’s tyrosine kinase (BTK) [2]. BTK is a 659 amino acid protein forming five signaling domains, which enable it to transmit and amplify signals from various cell surface receptors involved in the transmission of extracellular signals to intracellular signaling pathways [3]. As a non-receptor tyrosine kinase, BTK is expressed in most hematopoietic cells, especially in B cells, leading to the development and activation of B cells through B-cell antigen receptor (BCR) and Toll-like receptor (TLR) signaling [4–6]. BTK signaling contribute to the pathogenesis of autoimmune disease in synergy with TLR-mediated pathways [5, 7, 8]. Indeed, antigens bind to BCR and activate BTK, leading to phospholipase-Cγ (PLC-γ) signaling, which in turn activates the NF-κB and MAP kinase pathways, triggering the expression of CD40, CD86, and CD69 on B cells that promote B cell activation and proliferation [9–12]. BTK remarkably activates the BCR signaling pathway leading to differentiation of B cells into self-reactive B cells as seen in autoimmune diseases [13, 14]. BTK also activates innate immune cells, including macrophages, mast cells, basophils, and neutrophils [15].

While multiple nonsteroidal anti-inflammatory drugs (NSAIDs), corticosteroids, and biologics are available for the treatment of immune-mediated diseases, many patients still do not achieve disease remission with available agents. Rituximab leads to some degrees of disease control by targeting B cell-dependent pathways; that being the case, BTK inhibitors (BTKIs), which also target B-cell-related pathways might be useful as independent therapies or adjuncts to the current treatment options. In the absence of BTK, BCR signaling is insufficient to induce B cell differentiation into mature peripheral B cells, which leads to impaired proliferation of B cells, expression of activation markers, production of antibodies and cytokines, and defective immune responses against infections. BTK inhibition represents a promising therapeutic approach for the treatment of immune-mediated diseases, as it has shown remarkable efficacy in the treatment of B-cell malignancies such as chronic lymphocytic leukemia (CLL), marginal zone lymphoma (MZL), Waldenström macroglobulinemia (WM), mantle cell lymphoma (MCL), various B-cell lymphomas, and chronic graft-versus-host disease (GvHD) [16].

In comparison with chemo-immunotherapy, BTKIs increased the treatment efficacy of B cell malignancies in patients with high-risk features and showed a better tolerability in frail older patients [3]. As the BTKIs target the ATP-binding site, they are classified into three categories, namely covalent irreversible inhibitors, covalent reversible inhibitors, and non-covalent reversible inhibitors. The first covalent irreversible BTKI for the treatment of B-cell tumors, ibrutinib that binds covalently to the cysteine-481 binding site of BTK, was approved by the Food and Drug Administration (FDA) in 2013 and has brought a promising idea for the treatment of immune-mediated diseases. These reversible covalent inhibitors dissociate from common thiols while maintaining sustained inhibition of a protein with a conserved cysteine, providing selectivity [17]. Non-covalent BTKi inhibits BTK by different mechanisms to covalent BTKi such as blocking ATP binding site of BTK, forming the hydrogen bonds, or decreasing surface expression of B-cell activation markers, but not by binding to the C481 site on BTK [18]. Therefore, non-covalent BTKi is considered a potential alternative therapeutic option for patients who developed acquired resistance due to BTK C481 mutations.

Herein, the available evidence on the action mechanisms, efficacy, safety, and side effects of BTKIs is reviewed. Then, the recent data obtained from preclinical studies and clinical trials of BTKIs for the treatment of autoimmune diseases such as pemphigus vulgaris, pemphigus foliaceus, bullous pemphigoid, systemic lupus erythematosus, Sjögren’s disease, rheumatoid arthritis, systemic sclerosis, multiple sclerosis, myasthenia gravis, and inflammatory diseases such as psoriasis, chronic spontaneous urticaria, atopic dermatitis, and asthma are discussed. In addition, BTKIs-related complications and dermatological toxicity are reviewed.

## BTK signaling

**Fig. 1:**
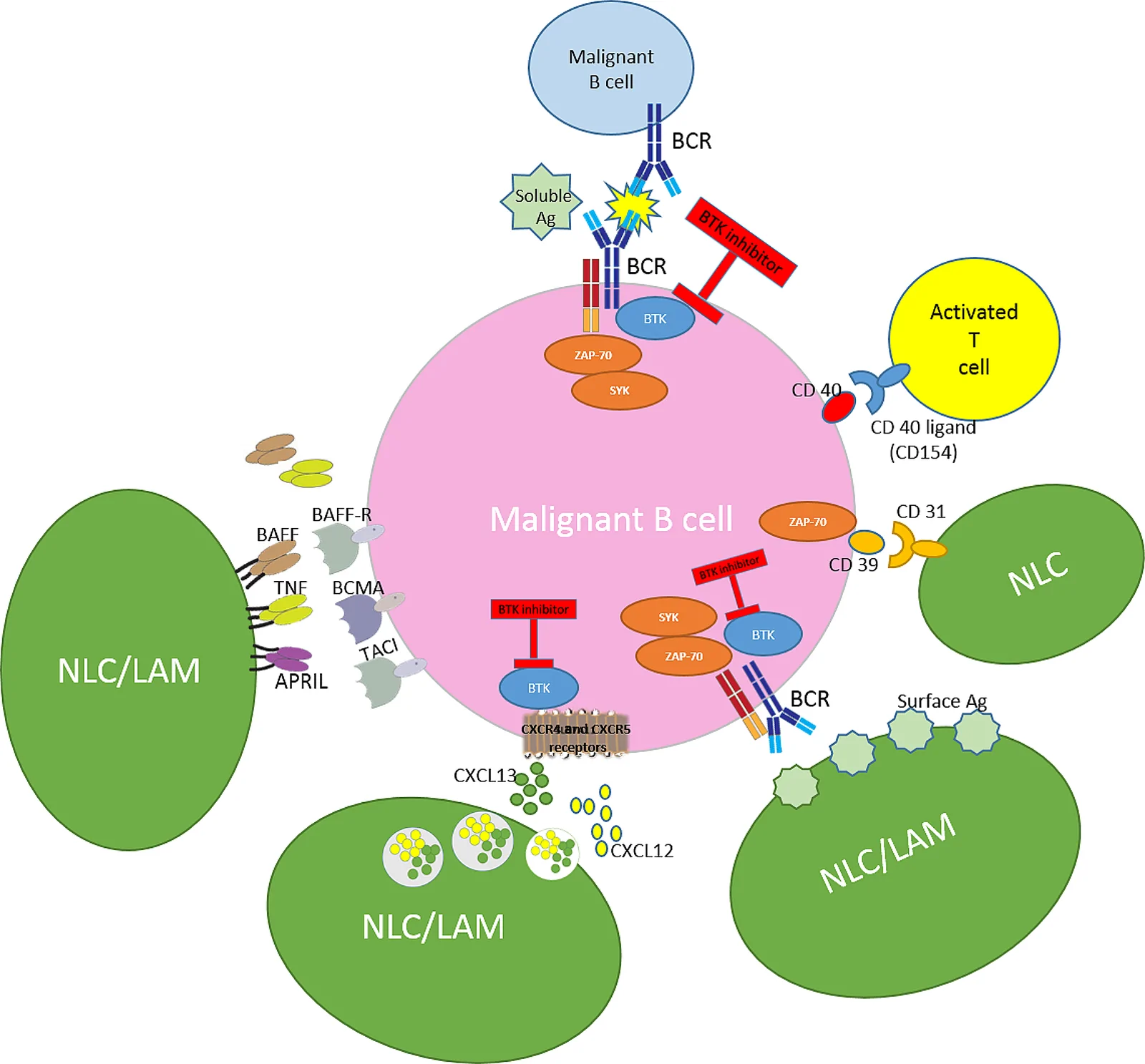
The figure discusses the interaction and signaling between NLCs, LAMs, and malignant B cells [19]. NLC/LAM expresses BAFF, APRIL, and TNF family members, which activate receptors on malignant B cells such as BAFF-R, BCMA, and TACI. Additionally, activated T helper cells express CD154 to interact with CD40, promoting the proliferation of malignant B cells. NLCs and stromal cells express CD31, which interacts with CD38 on malignant B cells, activating survival pathways. Cell-to-cell adhesion involves integrins and chemokine receptors, particularly VLA-4 integrins and chemokines CXCL12 and CXCL13. BTK is expressed by B cells, NLCs, and LAMs, contributing to signaling from other receptors and molecules

Malignant B cells such as CLL cells, MCL cells, and other stromal cells such as monocyte-derived nurse-like cells (NLC), called lymphoma-associated macrophages (LAM), and T lymphocytes reside in secondary lymphatic organs (i.e., lymph nodes, spleen, and tonsils) constituting the marrow or lymphoid tissue microenvironments [19] (Fig. 1). Chemokine receptors and adhesion molecules establish communication between cells in the microenvironment. Two pathways activate BCR signaling: (1) soluble or surface-bound antigens, (2) homotypic interactions of two BCR molecules. NLC/LAM express B cell-activating factor (BAFF), tumor necrosis factor (TNF) family members, and APRIL (also known as TNFSF13), which activate corresponding receptors on malignant B cells such as BAFF-receptor (BAFF-R), B cell maturation antigen (BCMA), and TACI (also known as TNFRSF13B) to trigger proliferation and survival signals. Activated T helper cells express CD40 ligand (CD154) on its surface to interact with CD40, leading to proliferation and growth of malignant B cells. NLCs and other stromal cells secrete chemokines, such as CXCL12 and CXCL13 and express CD31 on surface to interact with CD38 on the surface of malignant B cells. Activated CD38 engages with ZAP-70, resulting in downstream survival pathways. Cell-to-cell adhesion is established by integrins, particularly VLA-4 integrin (CD49d) on the surface of malignant B cells, and chemokine receptors. Stimulation of the BCR complex (BCR and CD79a, b) also activates SYK and ZAP-70. Stimulated NLC/LAM secret chemotactic factors such as chemokines CXCL12 and CXCL13 to attract malignant B cells such as CLL cells to the microenvironment via CXCR4 and CXCR5 receptors on the CLL cells. BTK is expressed by B cells, NLC, and LAM, contributing to the signaling of other surface receptors, such as CXCR4, CXCR5, and adhesion molecules (integrins).

## BTK as a therapeutic target

**Fig. 2:**
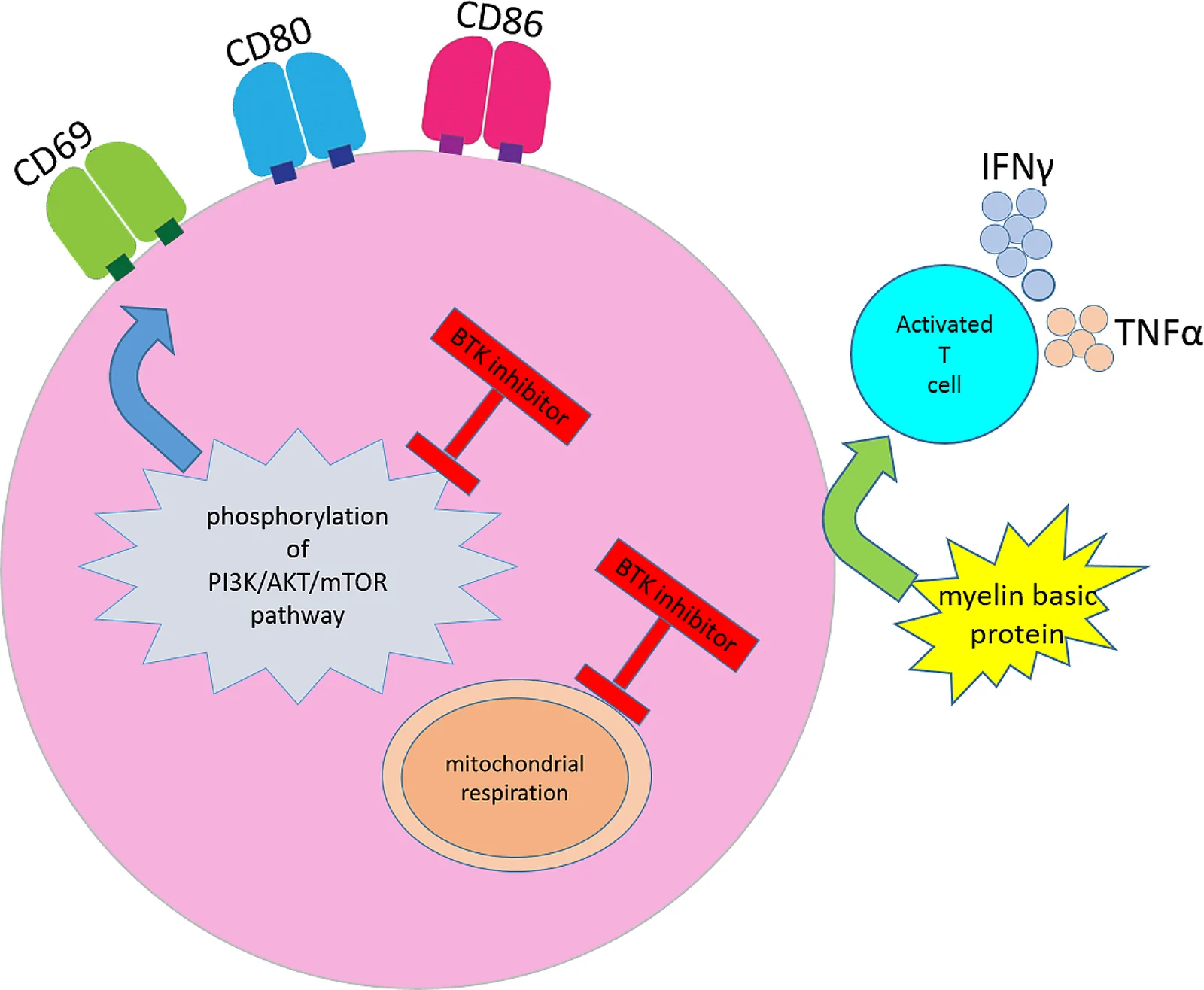
The BTK inhibitors limit the expression and upregulation of CD69 and co-stimulatory molecules CD80 and CD86 through the PI3K/AKT/mTOR pathway, which is responsible for B cell activation [20]. BTKi reduces AKT phosphorylation and activity, leading to decreased expression of CD80 and CD86 [20]. Moreover, BTKi decreases the polyclonal proliferation of CD8 + and CD4 + T cells and their production of pro-inflammatory cytokines [21]. It also modulates B cell metabolic processes by reducing mitochondrial respiration, which is important for B cell activation. These effects result in decreased B cell stimulation of T cells and limited expression of pro-inflammatory cytokines from both B cells and T cells

BTKIs target BCR signaling cascade (Fig. 2). As a result, BTKIs disrupt the B cells’ microenvironment, which explains the redistribution of lymphocytosis interactions in well-treated CLL patients [3]. To date, studies have shown that BTKIs limit the expression and upregulation of CD69, and co-stimulatory molecules such as CD80 and CD86 through the phosphatidylinositol 3 kinase (PI3K)/ protein kinase B (PKB, also known as AKT)/ mammalian target of rapamycin (mTOR) pathway, which all are responsible for the induction of B cell activation [20]. In detail, BTKIs decrease the phosphorylation of AKT and suppresses AKT activity. Inhibition of either AKT, PI3K, or mTOR pathways reduces the expression of co-stimulatory molecules such as CD80 and CD86 [20]. BTKIs also affect the B-T cells interactions by decreasing polyclonal proliferation of both CD8 + and CD4 + T cells and cellular expression of pro-inflammatory cytokines of Th17 and Th1, interferon gamma (IFN-γ), and TNF-α. BTKIs also modulate B cell metabolic processes through reducing B cell mitochondrial respiration, selectively for activated B cells. Since mitochondrial respiration is more important for B cell’s co-stimulatory molecule expression than glycolysis, inhibition of mitochondrial respiration leads to reduced B cell activation [20]. B cells stimulate T cells in response to myelin basic protein to secrete pro-inflammatory cytokines such as IFN-γ and TNF-α [21]. Hence, decreased activation of B cells reduces the stimulation of T cells. In conclusion, BTKIs regulate B cell mitochondrial metabolism and limit the expression of pro-inflammatory cytokines in both B and T cells.

## BTK inhibitors

In terms of mechanism of action, BTK inhibitors (BTKis) are classified into two main groups, reversible and irreversible BTKIs, based on the binding mode. Furthermore, based on the chemical interatomic linkage, BTKIs are classified into two groups, covalent and non-covalent BTKIs [22]. Covalent irreversible BTKIs bind cysteine 481 (C481) in the ATP-binding site of BTK by covalent irreversible bonds, resulting in blockage of the phosphorylation of downstream kinases in the BCR signaling pathway, thus blocking B cell activation. Covalent irreversible BTKIs consist of ibrutinib (Imbruvica), acalabrutinib (Calquence (ACP-196)), zanubrutinib (Brukinsa), evobrutinib, remibrutinib (LOU064), elsubrutinib, tolebrutinib (SAR442168), orelabrutinib, branebrutinib (BMS-986195), poseltinib, tirabrutinib hydrochloride (Velexbru) (GS-4059), spebrutinib (CC-292), SHR1459, TAS5315, AC0058TA, and BI-BTK-1. Ibrutinib is the first-generation of BTKIs and is approved to treat several B cell malignancies such as chronic lymphocytic leukemia (CLL), small lymphocytic lymphoma (SLL), Waldenström macroglobulinemia (WM), chronic graft versus host disease (GvHD) (after the failure of ≥ 1 lines of systemic therapy), mantle cell lymphoma (MCL) (after ≥ 1 prior therapy), and marginal zone lymphoma (MZL) (requiring systemic therapy and having received at least 1 prior anti-CD20-based therapy) [23]. Although the efficacy of ibrutinib is satisfying against the mentioned malignancies in clinics, off-target toxicities and drug resistance are reported. Thus, second-generation of BTKIs are developed such as acalabrutinib and zanubrutinib [24–26]. Non-covalent reversible inhibitors do not bind to the C481 site of BTK, but share the cysteine 481 binding site and are useful in patients with B cell malignancies resistant to prior therapy with covalent BTKIs. Despite being less advanced compared to irreversible BTKIs, reversible inhibitors have shown to be more effective for the treatment of autoimmune diseases, including rheumatoid arthritis, multiple sclerosis, systemic lupus erythematosus, and graft versus host disease (GvHD). Non-covalent reversible inhibitors consist of fenebrutinib (GDC-0853) and nemtabrutinib. Covalent reversible inhibitors consist of rilzabrutinib (PRN1008), BMS-986142, BIIB091. PRN473 (SAR444727) is both non-covalent and covalent reversible BTKI [6]. BTK inhibitors and their classifications are shown in Table 1.

**Table 1:** BTK inhibitors classification

BTKi classification	Mechanism of action	Inhibitors	
Covalent irreversible	Binding covalently to the cysteine-481 binding site of BTK by covalent irreversible bonds, resulting in blockage of the phosphorylation of downstream kinases in the BCR signaling pathway, thus blocking B cell activation	First-generation	Ibrutinib (Imbruvica)
		Second-generation	Acalabrutinib (Calquence (ACP-196)), Zanubrutinib (Brukinsa), Evobrutinib, Remibrutinib (LOU064), Elsubrutinib, Tolebrutinib (SAR442168), Orelabrutinib, Branebrutinib (BMS-986195), Poseltinib, Tirabrutinib hydrochloride (Velexbru) (GS-4059), Spebrutinib (CC-292), SHR1459, TAS5315, AC0058TA, and BI-BTK-1
Covalent reversible	Dissociating from common thiols while maintaining sustained inhibition of a protein with a conserved cysteine	Rilzabrutinib (PRN1008), BMS-986142, PRN473(SAR444727), and BIIB091	
Non-covalent reversible	Blocking ATP binding site of BTK, forming the hydrogen bonds, or decreasing surface expression of B-cell activation markers	Fenebrutinib (GDC-0853), Nemtabrutinib, and PRN473(SAR444727)	

## BTKI in autoimmune diseases

### Autoimmune blistering disorders

Pemphigus is an autoimmune disease characterized by painful blisters and erosions. In pemphigus, the immune system mistakenly attacks cells in the epidermis and the mucous membranes by immunoglobulin type G (IgG) autoantibodies against desmogleins, the adhesion proteins that bind keratinocytes to one another. When the bonds are disrupted, fluid collects between the epidermis layers, forming blisters. Pemphigus can be classified into two primary subtypes: pemphigus vulgaris (PV), in which blisters form in the mouth and other mucosal surfaces in addition to the skin and causes agonizing oral erosions, and pemphigus foliaceus (PF), which only affects the skin [27]. In pemphigus, activated T cells initiate an autoimmune cascade, which induces activated B lymphocytes to synthesize anti-desmoglein antibodies [28]. In pemphigus, activated neutrophils, eosinophils, and mast cells of the innate immune system accumulate in the lesions’ infiltrates [27]. Therefore, treatments have to target both adaptive and innate immune pathways. Although systemic corticosteroids are the mainstay of treatment (moderate to high doses of oral prednisone or prednisolone, or intravenous methylprednisolone), long time corticosteroid therapy may result in serious side effects such as gastritis, hypertension, diabetes mellitus, and osteoporosis. In bullous pemphigoid, IgG +/- IgE antibodies and activated T lymphocytes attack the basement membrane of the epidermis. The target is the protein BP180 (also known as type XVII collagen), or less frequently, BP230, a plakin. BP180 and BP230 are associated with the hemidesmosomes, structures that bind the epidermal keratinocytes to the dermis. Binding of the autoantibodies to proteins releases cytokines from T cells, leading to complement activation, recruitment of neutrophils, and release of proteolytic enzymes. Proteolytic enzymes destroy the hemidesmosomes and trigger the formation of subepidermal tense blisters. Most patients with bullous pemphigoid receive steroids, either prednisone or prednisolone. The dose is adjusted until the blisters and inflammatory lesions stop appearing, which usually takes several weeks. As mentioned earlier, systemic steroids have many undesirable side effects. Rilzabrutinib (PRN1008), a covalent reversible BTKI, combined with low doses of corticosteroid or as monotherapy is safe and efficient based on the clinical response in patients with pemphigus vulgaris [29]. A phase II trial of 27 patients with PV and PF showed promising results for using rilzabrutinib. More than half of the patients achieved disease control within 4 weeks without administration of prednisolone [30]. Furthermore, rilzabrutinib is granted Orphan Drug Designation by the United States Food and Drug Administration (FDA) for the treatment of PV (ANZCTR No. ACTRN12614000359639) [31]. Frequent mild gastrointestinal side effects were observed in rilzabrutinib therapy [31]. A phase III trial evaluated the efficacy and safety of oral rilzabrutinib in moderate to severe PV or pemphigus foliaceus (NCT03762265). It was reported that the proportion of patients meeting the primary endpoint on rilzabrutinib was not significantly different from placebo (NCT03762265) [32]. Rilzabrutinib in combination with corticosteroid was evaluated in another phase III clinical trial as a promising for its self-limited immunomodulatory effects for the treatment of newly diagnosed or relapsing PV; disease control was observed early and improved with continued treatment, and a favorable benefit-risk profile was achieved (NCT02704429) [33]. Regarding human and animal studies, rilzabrutinib has shown promising therapeutic results in humans with pemphigus, while PRN437 is more effective than rilzabrutinib in animal models with pemphigus [34]. Further case reports showed that ibrutinib, a covalent irreversible BTKI, could be administrated for the treatment of chronic lymphocytic leukemia (CLL) and acquired paraneoplastic pemphigus (PNP) [35, 36]. Tirabrutinib hydrochloride (Velexbru) (GS-4059) is a covalent irreversible BTKI, which reduces IgG production and impairs IgG autoantibody-mediated signaling pathway involved in the pathogenesis of pemphigus, thus could be an alternative therapy for resistant pemphigus [37]. Tirabrutinib is approved in Japan for the treatment of plasma cell lymphoma, Waldenström macroglobulinemia (WM), and primary lymphoma of the central nervous system. To evaluate the safety and efficacy of tirabrutinib, sixteen patients with refractory pemphigus were included in a phase II trial (JapicCTI-184231) [38]. Treatment with tirabrutinib caused remission in patients with refractory pemphigus and led to reduced oral corticosteroid exposure [38]. In in vivo studies, oral PRN473 that is both non-covalent and covalent reversible BTKi, is efficacious and well-tolerated in the treatment of canine pemphigus foliaceus (PF) [34, 39, 40]. Results of in vitro studies demonstrate that PRN473 is highly selective and has prolonged effect on BTK with minimal systemic effects [41]. The administered BTKIs in pemphigus are shown in Table 2.

**Table 2:** BTK inhibitors tested in preclinical and clinical trials in autoimmune blistering disorders

Disease	BTK inhibitor	Mechanism of action	Significant trial/study	Significant findings of clinical trial/study
Pemphigus vulgaris	Rilzabrutinib (PRN1008)	Second-generation covalent reversible, high affinity and selectivity for the BTK, anti-inflammatory effects	Phase II (in new-onset or relapsing, moderate‐to‐severe PV / healthy adult participants)	Safe and efficacious with rapid clinical activity and mild gastrointestinal system side effects in PV (NCT02704429 / ANZCTR No. ACTRN12614000359639) [30, 31]
			Phase III (in newly diagnosed or relapsing PV)	Early disease control and disease improvement with continued treatment, a favorable benefit-risk profile (NCT02704429) [32]
			Phase III (in moderate to severe PV or pemphigus foliaceus)	The proportion of patients meeting the primary endpoint on rilzabrutinib was not significantly different from placebo (NCT03762265) [33]
	Tirabrutinib	Second-generation covalent irreversible	Phase II (in refractory pemphigus)	The complete remission rate after 24-week treatment: 18.8% The cumulative complete remission rate after 52-week treatment: 50.0% (JapicCTI-184231) [38]
	Ibrutinib (PCI-32765)	First-generation covalent irreversible, off-target activity on EGFR, ErbB2, ITK, and TEC	Case-report (acquired paraneoplastic pemphigus)	A case report of PNP in the context of CLL, treated with ibrutinib [35]
			Case-report (acquired paraneoplastic pemphigus)	A case report of PNP in the context of B-CLL/SLL, treated with ibrutinib and rituximab [36]
Bullous pemphigoid	PRN473	Second-generation, both non-covalent and covalent reversible, anti-inflammatory effects in vitro and in vivo, very limited off-target effects	In vivo and in vitro study	Efficacious and well-tolerated in the treatment of canine pemphigus foliaceus (PF) [34, 39–41]

### Systemic lupus erythematosus

Systemic lupus erythematosus (SLE) is a multi-organ multi-factorial disease characterized by the autoreactive T and B cells and production of autoantibodies against self-antigens such as nucleic acids, DNA in both the double-stranded (Anti-dsDNA) and the single-stranded (Anti-ssDNA) conformations, RNA nuclear antigens such as the Ro/SAA, ribonucleoprotein, and non-nuclear components, and phospholipids. Indeed, autoantibodies progressively accumulate in tissues years before the clinical onset of SLE and form antigen-antibody complex deposits, causing inflammation and tissue injury [42, 43]. SLE flare is accompanied by an increase in autoantibodies (primarily anti-dsDNA) [44]. Therefore, B cell–targeting therapies can lead to B cell depletion, which is accompanied by a reduction in autoantibodies. Although corticosteroids and B cell–targeting therapies (monoclonal antibodies against CD20, CD19, and CD22) are essential components in SLE treatment, therapeutic outcomes are associated with severe side effects [45, 46]. Medications that inhibit more than one pathway in SLE pathogenesis would help to reach higher therapeutic efficacy. As mentioned earlier, BTK in B cells plays a key role in B cell activation and its differentiation; thus, targeting and depleting B cells via BTKI can be a viable alternative therapeutic modality. A study in the lupus nephritic mouse model showed that BTK inhibition dampened humoral autoimmunity [47]. Study on ibrutinib in lupus-prone B6.Sle1 or B6.Sle1.Sle3 mice revealed that humoral and cellular autoimmunity reduced; some autoantibodies, including anti­nucleosome antibodies and anti­histone antibodies, but not anti­dsDNA antibodies, reduced and led to improvement of lupus nephritis [48].

BI-BTK-1, a highly selective irreversible BTKI, is used to target both myeloid cell (particularly macrophage) and B cell activation and function in the MRL-lpr/lpr murine model of SLE. It is reported that lupus-associated cutaneous and neuropsychiatric disease decreased and cognitive function improved following reduced accumulation of macrophages, T cells, and B cells within the central nervous system, particularly the choroid plexus. Finally, skin lesions improved macroscopically and histologically in the mice model [49]. In a phase II trial of SLE, the efficacy and safety of fenebrutinib (GDC-0853), a non-covalent reversible BTKI, was assessed in 260 patients with moderate to severe SLE. Although levels of phosphorylated BTK, CD19 + B cells, autoantibodies (mainly anti­dsDNA antibodies) decreased and the BTK pathway was inhibited, fenebrutinib did not achieve a treatment benefit over the placebo group (NCT02908100) [50]. In another phase Ib/IIa trial, safety, tolerability, and preliminary efficacy of orelabrutinib (ICP-022) were evaluated. Orelabrutinib, a covalent irreversible BTKI, has shown to reduce levels of anti-dsDNA and IgG, total B cells, and increase C4 in patients with SLE. Orelabrutinib was generally safe and well tolerated in patients with mild to moderate SLE [51]. Zanubrutinib, a covalent irreversible BTKI, is in an ongoing phase II study to evaluate its efficacy in patients with active proliferative lupus nephritis (NCT04643470). Evobrutinib, a covalent irreversible BTKI, was evaluated in a phase II study for the efficacy and safety in patients with active autoantibody-positive SLE. It was reported that evobrutinib was not an effective therapeutic intervention for patients with SLE, but it was well tolerated at all doses, with no dose effect observed for treatment-emergent adverse event (NCT02975336) [52].

A phase II trial evaluated the safety and efficacy of elsubrutinib, a covalent irreversible BTKI, alone or in combination with upadacitinib (ABT-494), a JAK1 selective inhibitor, in patients with moderately to severely active SLE (NCT03978520). ABBV-599HD (Elsubrutinib 60 mg + upadacitinib 30 mg) resulted in significant improvements in SLE disease activity and reduced overall flares and time to first flares with acceptable safety through 48 weeks (NCT03978520) [53]. In another phase II trial, the safety of ABBV-599HD is being evaluated for adult patients with moderately to severely active SLE to assess change in disease state (NCT04451772).

Branebrutinib (BMS-986195), a covalent irreversible BTKI, was evaluated in a phase II trial for its safety and effectiveness in patients with active SLE; however, the data have not been published (NCT04186871). AC0058TA, a covalent irreversible BTKI, was evaluated for the safety, tolerability, pharmacokinetics, and pharmacodynamics in adult SLE patients with positive ANA levels in a phase Ib trial; however, no study results were posted or published about the clinical trial (NCT03878303). The administered BTKIs in SLE are shown in Table 3.

**Table 3:** BTK inhibitors tested in preclinical and clinical trials in systemic lupus erythematous

Disease	BTK inhibitor	Mechanism of action	Significant trial/study	Significant findings of clinical trial/study
Systemic lupus erythematous	Ibrutinib (PCI-32765)	First-generation covalent irreversible, off-target activity on EGFR, ErbB2, ITK, and TEC	lupus nephritic mouse model	Reduction in humoral and cellular autoimmunity and auto-Abs, including anti­nucleosome antibodies and anti­histone antibodies, but not anti­dsDNA antibodies. Lupus nephritis improved [48]
	BI-BTK-1	Second-generation irreversible, highly selective, and potent, targeting both macrophage and B cell activation	MRL-lpr/lpr murine model of SLE	Reduced lupus-associated cutaneous, neuropsychiatric disease phenotypes. Improved cognitive function via the reduction in the accumulation of T cells, B cells, and macrophages within the central nervous system (particularly the choroid plexus) in the MRL/lpr mice model. fewer skin lesions (macroscopically and histologically), reduced cutaneous cellular infiltration, and reduced inflammatory cytokine expression compared to control mice [49]
	Fenebrutinib (GDC-0853)	Second-generation non-covalent reversible, inhibits IgE-mediated histamine release from mast cells	Phase II (in moderate to severe active SLE)	Reduced levels of CD19 + B cells Reduced IgG anti-dsDNA autoantibodies, Did not achieve a treatment benefit over placebo group (NCT02908100) [50]
	Orelabrutinib (ICP-022)	Second-generation covalent irreversible	Phase Ib/IIa (in mild to moderate SLE)	Reduced levels of anti-dsDNA and IgG, total B cells, and increased complements C4 Safe and well tolerated in patients with mild to moderate SLE [51]
	Zanubrutinib (BGB-3111)	Second-generation covalent irreversible, lower off-target inhibitory activity on ITK, JAK3, and EGFR	Phase II (in active proliferative lupus nephritis)	Ongoing (NCT04643470)
	Evobrutinib (M2951)	Second-generation covalent irreversible both for BCR and Fc receptor signaling	Phase II (in autoantibody-positive SLE and active disease receiving SoC therapy)	All doses of evobrutinib were well tolerated, with no dose effect observed for treatment-emergent adverse event. Suggesting that BTK inhibition does not appear to be an effective therapeutic intervention for patients with SLE (NCT02975336) [52]
	Elsubrutinib	Second-generation covalent irreversible, inhibits histamine release from IgE-stimulated basophils and IL-6 release from IgG-stimulated monocytes	Phase II (in moderate to severe active SLE)	ABBV-599HD (Elsubrutinib 60 mg + upadacitinib 30 mg) led to significant improvements in SLE disease activity and reduced overall flares and time to first flares with acceptable safety through 48 weeks (NCT03978520) [53] Ongoing (NCT04451772)
	Branebrutinib (BMS-986195)	Second-generation covalent irreversible	Phase II (in active SLE)	The trial has been completed, but the data have not been published (NCT04186871)
	AC0058	Second-generation covalent irreversible, inhibits B-cell activation and inflammatory cytokine production in monocytes	Phase Ib	No data have been posted or published about the clinical trial (NCT03878303)

### Sjögren’s syndrome

Sjögren’s syndrome (SS) is a chronic inflammatory disease manifesting with dryness of the eyes (xerophthalmia), mouth (xerostomia), skin, mucosal surfaces, and extra-glandular involvement including arthritis, renal complications, vasculitis (mainly cryoglobulinemic vasculitis), and extranodal lymphoproliferation (causing lymphocytic interstitial pneumonitis) [54, 55]. The extraglandular manifestations of Sjögren’s syndrome are mainly associated with increase in auto-reactive B-cell stimulation, B-cell hyperactivity, increased levels of circulating immunoglobulins (autoantibodies), and alterations in B-cell subpopulations [56]. B cell–targeting therapies such as monoclonal antibodies against CD20 (such as rituximab) did not reach remarkable results in patients with Sjögren’s syndrome in two clinical trials [57, 58]. Remibrutinib (LOU064), a covalent irreversible BTKI, provides an alternative therapy for diseases driven by B cells, mast cells, and basophils such as Sjögren’s syndrome and has been assessed for its safety and tolerability in a phase I trial [59]. To assess basophil suppression by remibrutinib, CD203c inhibition was applied twice daily showing positive outcomes [60]. Remibrutinib (LOU064) has fewer side effects, higher specificity and potency of blocking activity than its ancestor molecules [61]. Remibrutinib is well-tolerated at all doses without any dose-limiting toxicity and demonstrates a safe profile and strong BTK inhibition in blood and skin pharmacodynamics in healthy human subjects and in healthy subjects with asymptomatic atopic diathesis [59]. Remibrutinib was evaluated in a phase II trial for its efficacy in patients with Sjögren’s syndrome, the trial has been terminated, but the data have not been published yet (NCT04035668). Tirabrutinib has been evaluated for its efficacy and safety in patients with moderate to severe active Sjögren’s syndrome, either primary or associated with a concomitant systemic autoimmune disease, through a phase II study (NCT03100942) [62]. Tirabrutinib demonstrated no significant differences versus placebo in primary and secondary endpoints [62]. Branebrutinib (BMS-986195) is well-tolerated and safe enough to be administrated in healthy humans in a phase I study [63]. Branebrutinib is rapidly absorbed with 100% occupancy of BTK after a single dose and inactivates BTK rapidly (NCT02705989) [63]. Branebrutinib was evaluated in a phase II trial to assess its safety and effectiveness in patients with primary active Sjögren’s syndrome; however, the data have not been published yet (NCT04186871). The administered BTKIs in Sjögren’s syndrome are shown in Table 4.

**Table 4:** BTK inhibitors tested in preclinical and clinical trials in Sjögren’s syndrome

Disease	BTK inhibitor	Mechanism of action	Significant trial/study	Significant findings of clinical trial/study
Sjögren’s syndrome	Remibrutinib (LOU064)	Second-generation covalent irreversible, TEC inhibitor in vitro, dependent platelet activation	Phase I (in healthy subjects and in healthy subjects with asymptomatic atopic diathesis)	Well-tolerated without any dose-limiting toxicity Demonstrated a safe profile and strong BTK inhibition in blood and skin pharmacodynamics in healthy (NCT03918980) [59]
			Phase II (in moderate to severe SS)	The trial has been terminated, and the data have not been published (NCT04035668)
	Tirabrutinib	Second-generation covalent irreversible	Phase II (in moderate to severe active SS, either primary or associated with a concomitant systemic autoimmune disease)	No significant differences versus placebo in primary and secondary endpoints (NCT03100942) [62]
	Branebrutinib (BMS-986195)	Second-generation a covalent irreversible	Phase I (in healthy humans)	A rapid and high occupancy of BTK Safe and well-tolerated (NCT02705989) [63]
			Phase II (in primary SS)	The trial has been completed, but the data have not been published (NCT04186871)

### Rheumatoid arthritis

Rheumatoid Arthritis (RA) is an autoimmune and inflammatory disease, in which the immune system attacks healthy cells causing inflammation (painful swelling) in the affected tissues [64]. In detail, RA involves dysregulated T and B lymphocyte proliferation, particularly B cells, via BCR signaling leading to the production of autoantibodies and inflammatory cytokines [65]. As mentioned earlier, BTK is expressed in myeloid cells, including neutrophils, mast cells, monocytes, and macrophages infiltrating into synovium in RA [66, 67]. Immune complexes containing IgG are present in the joints, affecting synovial macrophages to produce cytokines and matrix metalloproteinases (MMPs) that contribute to RA pathophysiology [65]. In addition, BTK mediates bone resorption by stimulating osteoclast proliferation and differentiation as a contributor to RA [65, 68]. Due to the significant role of BTK in the pathogenic pathways of RA, BTKIs could be promising options. Ibrutinib demonstrated good efficacy with antiarthritic activity in arthritic DBA/1 mice models [69]. By longitudinal integrative whole-exome, whole-transcriptome sequencing and targeted sequencing, it has been reported that the Long-term need for ibrutinib to treat chronic RA leads to development of acquired resistance in patients, particularly developing a C481S mutation, which promotes BTK activation [70]. Thus, ibrutinib can cause a potential problem for all BTKIs, reversible or irreversible, which target Cys-481 in patients with mantle cell lymphoma with acquired resistance to ibrutinib [70]. Spebrutinib (CC-292), a covalent irreversible BTKI, has demonstrated sustained BTK occupancy, with low, even undetectable plasma levels of the drug in healthy humans in a preliminary phase I trial, and occupied all circulating BTK protein [11]. However, spebrutinib did not achieve significant clinical efficacy in a phase IIa trial in active RA patients on background methotrexate therapy (NCT01975610) [71]. Despite the lack of clinical efficacy in the trial, patients treated with spebrutinib showed a statistical reduction in chemokines CXCL13 and MIP-1β (implicated in B cell trafficking) and serum CTX-I (a measure of osteoclastic activity) compared to placebo [71]. Acalabrutinib (ACP-196), second-generation covalent irreversible BTKI, was also assessed in a phase IIa trial in 31 active RA patients on background methotrexate but did not show a meaningful clinical response after 4 weeks of treatment (NCT02387762). Fenebrutinib (GDC-0853) has demonstrated proper efficacy in a phase II trial in older/unfit patients and those with high-risk and/or relapsed CLL [25]. The primary outcome of higher doses of fenebrutinib (150–200 mg twice daily) was more than 50% clinical improvement (ACR50) compared to placebo, at 12 weeks of treatment according to the American College of Rheumatology criteria [25]. Poseltinib (LY3337641/HM71224), a novel BTKI acting on B cell activation and osteoclast formation, is evaluated through in vitro studies. Poseltinib blocks phosphorylation of BTK, ERK, and PLCγ2 resulting in suppression of osteoclast formation and inhibition of the upregulation of activation markers such as CD40, CD86, and CD69 on stimulated B cells [72]. To evaluate the efficacy and safety of poseltinib, a phase II trial was designed which was terminated after interim data did not demonstrate significant efficacy with no difference between doses of poseltinib and placebo at week 12 in moderate-to-severe RA patients (NCT02628028) [73]. BMS-986142, a covalent reversible BTKI, was evaluated for safety and efficacy in patients with moderate to severe RA with an inadequate response to methotrexate alone or methotrexate with up to 2 TNF Inhibitors, but the data have not been published (NCT02638948). Evobrutinib, a covalent irreversible BTKI, was assessed to determine efficacy, dose-response, and safety in active moderate to severe RA with previous methotrexate treatment, and it was well tolerated across indications at all doses (NCT03233230) [74]. Tirabrutinib (GS-4059) is evaluated for its safety profile, tolerability, and effect on disease-specific clinical markers and outcomes in patients with RA in a phase I trial [75]. Patients who received tirabrutinib 20 mg daily for four weeks achieved ACR20 in 38% of patient group compared to 20% for the placebo group, up to one month after treatment [75]. The safety and efficacy of elsubrutinib were evaluated on a background of upadacitinib, conventional synthetic disease-modifying anti-rheumatic drugs (csDMARDs), in a phase II trial to define the optimal dose for further development in patients with rheumatoid arthritis and inadequate response or intolerance to biological disease-modifying antirheumatic drugs; and it was reported that significant improvements in disease activity metrics of RA was achieved (NCT03682705) [76]. Branebrutinib (BMS-986195) was investigated in a phase I trial to evaluate the effects in healthy male subjects and in patients with moderate to severe RA; however, the trial was terminated without reporting the results (NCT03245515). Another phase I trial was conducted to evaluate the effects in healthy male subjects and in patients with moderate to severe RA and the trial has been completed, but the data have not been published (NCT02638948). Another phase I trial was conducted to assess the effect of branebrutinib on the pharmacokinetics of methotrexate, caffeine, montelukast, flurbiprofen, omeprazole, midazolam, digoxin, and pravastatin; the trial has been completed, but the data have not been published (NCT03131973). TAS5315, an irreversible covalent BTKI, was evaluated in a phase II trial to assess the efficacy and safety of TAS5315 in combination with methotrexate in 12 weeks or 36 weeks in patients with moderate to severe RA with inadequate response to maximally tolerated methotrexate dose, and it was reported that some bleeding risks occured, and nevertheless demonstrated numerical differences, compared with placebo, in the improvement rates of all measures of RA disease activity (NCT03605251) [77]. The administered BTKIs in RA are shown in Table 5.

**Table 5:** BTK inhibitors tested in preclinical and clinical trials in rheumatoid arthritis

Disease	BTK inhibitor	Mechanism of action	Significant trial/study	Significant findings of clinical trial/study
Rheumatoid arthritis	Ibrutinib (PCI-32765)	First-generation covalent irreversible, off-target activity on EGFR, ErbB2, ITK, and TEC	arthritic DBA/1 mice models	Efficacious with antiarthritic activity [69]
			longitudinal integrative whole-exome, whole-transcriptome sequencing and targeted sequencing (in patients with mantle cell lymphoma with acquired resistance to ibrutinib)	C481S mutation in long-term use of ibrutinib, acquired resistance to BTKi [70]
	Spebrutinib (CC-292)	Second-generation covalent irreversible, highly selective BTKi, near-complete BTK occupancy for 8–24 h	Phase I (in healthy humans)	Engaged all circulating BTK protein, undetectable plasma levels of the drug [11]
			Phase IIa (in active RA on background methotrexate therapy)	Did not achieve a significant efficacy, but a reduction in chemokines CXCL13 and MIP-1β and serum CTX-I (NCT01975610) [71]
	Acalabrutinib (ACP-196, trade name: Calquence)	Second-generation covalent irreversible	Phase IIa (in active RA on background methotrexate)	Did not show a meaningful clinical response after four weeks of treatment (NCT02387762)
	Fenebrutinib (GDC-0853)	Second-generation non-covalent reversible, IgE-mediated histamine release from mast cells	Phase II (in older/unfit patients and those with high-risk and/or relapsed CLL)	The primary outcome of higher doses (150–200 mg twice daily) was more than 50% clinical improvement (ACR50) compared to placebo, at 12 weeks of treatment [25]
	Poseltinib (LY3337641/HM71224)	Second-generation non-covalent irreversible, blocking phosphorylation of BTK, ERK, and PLCγ2 Inhibition of upregulation of the activation markers such as CD40, CD86, and CD69 on stimulated B cells	In vitro	Inhibition of B cell activation and osteoclast formation [72]
			Phase II (in moderate-to-severe RA)	Interim data did not demonstrate significant efficacy with no difference between doses of poseltinib and placebo at week 12 in RA patients (NCT02628028) [73]
	BMS-986142	Second-generation covalent reversible, reduced FcR-mediated cytokine production and BCR-induced cytokine production	Phase II (in moderate to severe RA)	The trial has been completed, but the data have not been published (NCT02638948)
	Evobrutinib (M52951)	Second-generation covalent irreversible	Phase IIb (in active moderate to severe RA with previous methotrexate treatment)	Generally well tolerated across indications at all doses (NCT03233230) [74]
	Tirabrutinib (GS-4059)	Second-generation covalent irreversible	Phase I	Patients who received 20 mg daily for four weeks achieved ACR20 in 38% of patients up to one month after treatment compared to 20% for the placebo [75]
	Elsubrutinib	Second-generation covalent irreversible, inhibits histamine release from IgE-stimulated basophils and IL-6 release from IgG-stimulated monocytes	Phase II (in patients with RA and inadequate response or intolerance to biological disease-modifying antirheumatic drugs)	Elsubrutinib in combination of Upadacitinib (ABBV-599): Significant improvements in disease activity metrics of RA (NCT03682705) [76]
	Branebrutinib (BMS-986195)	Second-generation covalent irreversible, inhibits histamine release from IgE-stimulated basophils and IL-6 release from IgG-stimulated monocytes	Phase I (in healthy male subjects)	Terminated without reporting the results (NCT03245515)
			Phase I (in moderate to severe RA)	The trial has been completed, but the data have not been published (NCT02638948)
			Phase I	The trial has been completed, but the data have not been published (NCT03131973)
	TAS5315	Second-generation covalent irreversible, highly selective BTKi with Cys481	Phase IIa (in moderate-to-severe RA with inadequate response to maximally tolerated methotrexate doses)	Demonstrating some bleeding risks, but nevertheless demonstrated numerical differences, compared with placebo, in the improvement rates of all measures of RA disease activity (NCT03605251) [77]

### Systemic sclerosis

Systemic sclerosis (SSc) is an autoimmune disorder, in which the immune system attacks the connective tissue of the skin, internal organs, and blood vessels resulting in fibrosis formation [78]. Pulmonary and cardiac fibrosis and particularly pulmonary hypertension are severe fatal complications [79, 80]. Accumulating evidence suggests that impaired function of regulatory and effector B cells leads to immune dysregulation, hyperreactivity, and chronic activation of effector B cells, which in turn, increases the production of autoantibodies, vasculopathy, and chronic activation of fibroblasts [81, 82]. Moreover, B cell-derived profibrotic IL-6 and TNF-α in response to TLR9 stimulation contribute to the pathogenesis of SSc [83]. Tocilizumab (Actemra), an IL-6-receptor-α inhibitor, failed to reduce skin thickening but caused modification of Rodnan Skin Score and improved pulmonary function in a phase III study [84]. Immunomodulators addressing B cells such as rituximab and tocilizumab in patients with SSc showed mixed efficacy with complete B cell depletion in a case-control study [84, 85]. Ibrutinib was assessed in 24 patients with SSc and showed suppressed production of the profibrotic cytokines IL-6 and TNF-α of effector B cells and also less activated phosphorylated NF-κB in an in vitro model of SSc sample [81]. In addition, autologous stem cell transplantation is an available treatment but only for selective patients with severe disease and high risk of major organ failure. The administered BTKIs in SS are shown in Table 6.

**Table 6:** BTK inhibitors tested in preclinical and clinical trials in systemic sclerosis, multiple sclerosis, and myasthenia gravis

Disease	BTK inhibitor	Mechanism of action	Significant trial/study	Significant findings of clinical trial/study
Systemic sclerosis	Ibrutinib (PCI-32765)	First-generation covalent irreversible, off-target activity on EGFR, ErbB2, ITK, and TEC	In vitro	Reduction in production of the profibrotic cytokines IL-6 and TNF-α Less activated phosphorylated NFκB [81]
Multiple sclerosis	Tolebrutinib (SAR442168)	Second-generation covalent irreversible CNS-penetrant Immunomodulatory activities	Phase IIb (in relapsing-remitting MS or relapsing secondary progressive MS)	Reduction in the number of gadolinium-enhancing lesions in relapsing MS after 12 weeks of treatment (NCT03889639) [86]
			Phase II (in patients with chronic MS)	None of the paramagnetic rim lesions (PRLs) had disappeared, no effect on smoldering inflammation, the study has been hold due to liver damage (NCT04742400) [87]
			Phase III (in nonrelapsing in primary progressive MS (PPMS))	Ongoing (NCT04458051)
			Phase III (in nonrelapsing in secondary progressive MS)	The study has been hold due to liver damage (NCT04411641)
			Phase III (in relapsing MS)	Ongoing (NCT04410991)
	Evobrutinib	Second-generation oral covalent irreversible, both for BCR and Fc receptor signaling	Phase II (in patients with relapsing MS)	Receiving 75 mg once daily: Fewer lesions than those receiving placebo after 12 weeks of treatment receiving 25 mg once daily or 75 mg twice daily: Did not show any significant difference versus placebo (NCT02975349) [88]
			Phase III (in patients with relapsing MS)	Evobrutinib administered orally twice daily versus teriflunomide (Aubagio) administered orally once daily: Did not lead to a more superior reduction in annualized relapse rates than teriflunomide (NCT04338022)
	Orelabrutinib (ICP-022)	Second-generation a covalent irreversible	Phase II (in patients with relapsing-remitting MS (RRMS))	Significant reductions in new active brain lesions (NCT04711148)
	BIIB091	Second-generation reversible selective, small-molecule	In vivo	Inhibition of B-cell activation and auto-Abs production [89]
			Phase I (in healthy volunteer)	Inhibition of naïve and memory B-cell activation without change in myeloid or lymphoid cell survival after 14 days of dosing [89]
	Fenebrutinib (GDC-0853)	Second-generation non-covalent reversible, inhibits IgE-mediated histamine release from mast cells	Phase III (in adult patients with relapsing-remitting MS)	Ongoing (NCT04586023)
Myasthenia gravis	Tolebrutinib (SAR442168)	Second-generation covalent irreversible, CNS-penetrant Immunomodulatory activities	Phase III (in adult patients with moderate-to-severe generalized MG)	Terminated due to the drug-induced liver injury (NCT05132569)

### Multiple sclerosis

Multiple sclerosis (MS) is an autoimmune disease, in which the immune system attacks the myelin sheath of neurons, resulting in slowed and disrupted nerves’ conduction. In the cerebrospinal fluid of MS patients, a significant increase in the expression of B cell co-stimulatory molecules such as CD80 and CD86 is observed [21]. B cells stimulate T cells in response to myelin basic protein to secrete pro-inflammatory cytokines such as IFN-γ and TNF-α [20, 21]. BTK is expressed in microglia (myeloid cells) and B cells of the central nervous system. As mentioned earlier, BTKI reduces B cells’ mitochondrial respiration; thus treatment with BTKI can be considered as therapeutic agents in patients with MS [20]. Tolebrutinib (SAR442168) that crosses the blood-brain barrier is a covalent irreversible BTKI, which was assessed in a phase IIb trial for its efficacy and safety in relapsing-remitting MS or relapsing secondary progressive MS [86]. Reduction in the number of gadolinium-enhancing lesions was reported after 12 weeks of treatment (NCT03889639) [86]. In another phase II trial, tolebrutinib at a 60 mg daily dose for 48 weeks (nearly one year) was evaluated to see if it could help clear chronically inflamed brain’s white matter lesions in MS (NCT04742400) [87]. None of the paramagnetic rim lesions (PRLs) had disappeared despite nearly a year of treatment, suggesting that tolebrutinib had no effect on smoldering inflammation [87]. Since the patients needed to transition to the higher dose of 120 mg daily for the next 48 weeks and the higher dose of tolebrutinib might lead to liver damage, the study had been hold [87]. In a phase III trials, tolebrutinib is currently being evaluated in delaying disability progression in nonrelapsing in primary progressive MS (PPMS) to determine the efficacy, safety, tolerability, pharmacokinetics, pharmacodynamics, and the efficacy on clinical endpoints, magnetic resonance imaging (MRI) lesions, cognitive performance, physical function, and patient’s quality of life (NCT04458051). Moreover, in another phase III trials, tolebrutinib was evaluated in delaying disability progression in nonrelapsing in secondary progressive MS (NRSPMS) (NCT04411641). However, the trial was placed on hold because of reported cases of drug-induced liver injury in patients, potentially caused by a preexisting factors related to hepatic dysfunction (NCT04411641). In another phase III trial in patients with relapsing MS, efficacy, safety, tolerability, and pharmacodynamics of daily tolebrutinib is being assessed compared to teriflunomide (Aubagio) on disability progression, MRI lesions, cognitive performance, and quality of life (NCT04410991). Evobrutinib that affects B cell activation both in vitro and in vivo, was assessed in a phase II trial in patients with relapsing MS. Patients who received 75 mg of evobrutinib once daily had fewer lesions than those receiving placebo after 12 weeks of treatment. However, patients who received 25 mg once daily or 75 mg twice daily did not show any significant difference versus placebo. Longer and larger trials are necessary to assess the efficacy of evobrutinib [88]. A phase III trial evaluated the efficacy and safety of evobrutinib administered orally twice daily versus teriflunomide (Aubagio) administered orally once daily in patients with relapsing MS (NCT04338022). However, results of the trial revealed that evobrutinib did not lead to a more superior reduction in annualized relapse rates than teriflunomide (NCT04338022). Orelabrutinib in a phase II trial was evaluated to detect the number of new brain lesions with active inflammation after 12 weeks and also its efficacy, safety, and relapse rates after 120 weeks (NCT04711148) and led to significant reductions in new active brain lesions among patients with relapsing-remitting MS (RRMS) (NCT04711148). BIIB091, a novel selective covalent reversible small-molecule BTKI, has been evaluated in vivo and in a phase I trial so far. In in vivo studies, BIIB091 inhibited B cell activation and autoantibodies production [89]. In a phase I trial, BIIB091 inhibited naïve and memory B cell activation with a minor impact on myeloid or lymphoid cell survival after 14 days of dosing in healthy volunteers [89]. Fenebrutinib is currently in an ongoing phase III clinical trial for evaluation of its efficacy and safety on disability progression and relapse rate in adult participants with PRMS (NCT04586023). The administered BTKIs in MS are shown in Table 6.

### Myasthenia gravis

Myasthenia gravis (MG) is a chronic autoimmune neuromuscular disease that causes weakness in the skeletal muscles such as arms, legs, and breathing muscles worsening after periods of activity and improving after periods of rest. In MG, antibodies against acetylcholine receptors (AChR), muscle-specific kinase (MuSK), and lipoprotein receptor related protein 4 (LRP4) block and destroy the receptors at the neuromuscular junction [90, 91]. Circulating antibodies against AChR are detected in blood samples of most MG patients [92]. Interaction between activated T and B cells leads to the production of IgG-type antibodies [93]. BTKI may be a promising therapeutic option for the treatment of MG; however, there has been only one clinical trial to assess the effect of BTKIs on MG patients. Tolebrutinib was assessed in phase III trial to evaluate its efficacy and safety in adult patients with moderate-to-severe generalized MG (NCT05132569). The trial was terminated due to the drug-induced liver injury in patients, potentially caused by a preexisting factors related to hepatic dysfunction (NCT05132569). The administered BTKIs in MG are shown in Table 6.

## BTKI in inflammatory diseases

### Psoriasis

Psoriasis is characterized by raised plaques and scales on the skin caused by dysfunction of the immune system [94]. Indeed, an overactive immune system speeds up skin cell growth, thus skin epithelial keratinocytes pile up forming pink or red patches, and white or silvery scales [95]. Oxidative stress is known as an important contributor to the pathogenesis of psoriasis. Neutrophils secrete oxidants through the BTK pathway that maintains inflammation in psoriasis [96, 97]. In addition, BTK executes signaling functions in dendritic cells and γδ + T cells. In detail, activation of the BTK pathway upregulates inflammatory cytokines such as IL-23/TNF-α in dermal CD11c dendritic cells and IL-17 A in γδ + T cells. Ibrutinib was evaluated in dermal psoriasis-like inflammation of the imiquimod-induced (IMQ) mouse model [98]. Preventive treatment with ibrutinib in the IMQ mouse model led to the reduction in IL-23/TNF-α levels of CD11c dendritic cells and IL-17 A levels of γδ + T cells; thus, ibrutinib reduces oxidative stress in these innate immune cells, which makes a promising therapeutic option for psoriasis [37]. The administered BTKIs in psoriasis are shown in Table 7.

**Table 7:** BTK inhibitors tested in preclinical and clinical trials in psoriasis, chronic spontaneous urticaria, atopic dermatitis, and asthma

Disease	BTK inhibitor	Mechanism of action	Significant trial/study	Significant findings of clinical trial/study
Psoriasis	Ibrutinib (PCI-32765)	First-generation covalent irreversible, off-target activity on EGFR, ErbB2, ITK, and TEC	Imiquimod-induced (IMQ) mouse model	Reduction in IL-23/TNFα levels of CD11c dendritic cells and IL-17 A levels of γδ + T cells [37]
Chronic spontaneous urticaria	Fenebrutinib (GDC-0853)	Second-generation non-covalent reversible, inhibits IgE-mediated histamine release from mast cells	Phase II (in adult patients with CSU for more than six months and symptomatic despite treatment with H1 antihistamines (up to fourfold the approved dose))	Good efficacy with the reduction in IgG-anti-FcεRI auto-Abs at week 8 at all dose levels compared to placebo, terminated due to the transient transaminase elevations in a limited number of patients and safety issues (NCT03137069) [100]
	Remibrutinib (LOU064)	Second-generation covalent irreversible, TEC inhibitor in vitro, dependent platelet activation	Phase I (in healthy subjects with asymptomatic atopic diathesis)	Near complete basophil or skin prick test (SPT) inhibition at greater than or equal to 50 mg q.d. for CD63 and at greater than or equal to 100 mg q.d, Well-tolerated at all doses without any dose‐limiting toxicity with a favorable safety profile (NCT03918980) [59]
			Phase III (patient with CSU with inadequately controlled by H1 antihistamines)	Ongoing (NCT05030311)
	Rilzabrutinib (SAR444671)	Second-generation covalent reversible, anti-inflammatory effects	Phase II (in symptomatic CSU patients despite the use of H1 antihistamine)	Ongoing (NCT05107115)
Atopic dermatitis	PRN437 (SAR444727)	Second-generation both non-covalent and covalent reversible, topically administered Inhibition of neutrophil migration and monocyte activation mediated by IgG (FcgR) and mast cell and basophil activation mediated by IgE (FceR) Inhibition of the β2-integrin c-1 activation and subsequently neutrophil recruitment into inflamed tissue	Phase IIa (mild to moderate AD)	The trial has been completed, but the data have not been published (NCT04992546)
	Ibrutinib (PCI-32765)	First-generation covalent irreversible, off-target activity on EGFR, ErbB2, ITK, and TEC	Phase III (in patients with previous allergic reactions to peanut and/or tree nuts)	Eliminating aeroallergen skin test and BTKi, suppressing IgE-mediated basophil activation in adults suffering from allergy to peanut or tree nut (NCT03149315) [107]
	Branebrutinib (BMS-986166)	Second-generation covalent irreversible, inhibits histamine release from IgE-stimulated basophils and IL-6 release from IgG-stimulated monocytes	Phase II (moderate to severe AD)	The trial has been completed, but the data have not been published (NCT05014438)
Asthma	Rilzabrutinib (PRN1008)	Second-generation covalent reversible, anti-inflammatory effects	Phase II (moderate-to-severe asthma)	Ongoing (NCT05104892)

### Chronic spontaneous urticaria

Chronic spontaneous urticaria (CSU), also known as chronic idiopathic urticaria, is the presence of urticaria (hives) on most days of the week, for a duration of six weeks or longer. Mechanistically, CSU happens because of infiltration of mainly T helper 2 cells (Th2) around small venules of the skin [99]. BTK is required in the activation of mast cells via FcεRI and producing autoantibodies by B cells. Thus, BTKI might be effective in CSU. Fenebrutinib (GDC-0853) was assessed in a phase II trial in adult patients with CSU for more than six months and symptomatic despite treatment with H1 antihistamines (up to fourfold the approved dose); IgG-anti-FcεRI autoantibodies significantly decreased at week 8 at all dose levels compared to placebo, which demonstrated good efficacy in patients with CSU, but the long-term extension of the trial was also terminated due to the transient transaminase elevations in a limited number of patients and safety issues (NCT03137069) [100]. Notably, fenebrutinib did not result in remarkable reductions in IgG subtypes such as IgG1 and IgG3 [101]. Remibrutinib (LOU064) provides an alternative therapy for diseases driven by B cells, mast cells, and basophils such as CSU. Remibrutinib was evaluated for its clinical safety and pharmacodynamics in CSU with asymptomatic atopic diathesis in a phase I clinical trial (NCT03918980) [59]. Remibrutinib was well-tolerated at all doses without any dose‐limiting toxicity with a favorable safety profile and near complete basophil or skin prick test (SPT) inhibition was achieved at greater than or equal to 50 mg q.d. for CD63 and at greater than or equal to 100 mg q.d [59]. An ongoing phase III trial is designed to evaluate the efficacy and safety of remibrutinib in the treatment of CSU in adults which was inadequately controlled by H1 antihistamines (NCT05030311). Rilzabrutinib (SAR444671) is currently being assessed in an ongoing phase II trial for the safety and effectiveness of 3 oral doses, compared with placebo for decreasing the frequency and severity of pruritus and urticaria in patients with CSU (NCT05107115). The administered BTKIs in CSU are shown in Table 7.

### Atopic dermatitis

Atopic dermatitis (AD), the most common form of eczema, is a chronic inflammatory disorder causing dry, itchy, inflamed, and cracked skin [102]. AD is usually a chronic condition and common in young children but also occurs at any age [103, 104]. PRN437 (SAR444727), both non-covalent and covalent reversible topically administered BTKI, inhibits three pathways including the activation of monocyte and neutrophil migration mediated by IgG (FcgR), the activation of mast cell and basophil mediated by IgE (FceR), and the activation of the β2-integrin c-1 and subsequently neutrophil recruitment into inflamed tissue [105, 106]. PRN473 (SAR444727) was evaluated for the safety, tolerability, and efficacy in phase IIa in 40 patients with mild to moderate AD; the trial has been completed, but the data have not been published (NCT04992546). It is reported in a study that ibrutinib (PCI-32765) therapy suppresses IgE-mediated basophil activation and reduces mast cell and basophil reactivity to the allergens in adults suffering from allergy to peanut or tree nut (NCT03149315) [107]; therefore, ibrutinib eliminates aeroallergen skin test [107, 108]. Branebrutinib (BMS-986166) was evaluated in phase II trial to evaluate the efficacy, safety, and tolerability for the treatment of patients with moderate to severe AD; the trial has been completed, but the data have not been published (NCT05014438). The administered BTKIs in AD are shown in Table 7.

### Asthma

Asthma is associated with chronic inflammation, airway hyper-responsiveness, and reversible airflow obstruction. Accumulation of inflammatory mediators, cytokines, chemokines, infiltrating immune cells in airways leading to remodeling of the airways, including subepithelial fibrosis, myofibroblast hyperplasia, goblet cell hyperplasia, wall thickening, smooth muscle cell hyperplasia and hypertrophy, epithelial hypertrophy, and airway wall thickening [109–111]. B cells produce antibodies such as IgE [112]. IgE binds to its receptor (FcqRII or CD23) and induces CD23-mediated eosinophilic infiltration causing airway hyper-responsiveness of asthma [113]. Stimulation of tyrosine kinases such as SYK, ZAP-70, BTK, and ITK on B cells is the earliest signaling response in inflammatory cells; thus, BTKI can be a therapeutic option for asthma. Rilzabrutinib is being assessed in an ongoing phase II trial to evaluate its efficacy, safety, and tolerability in patients with moderate-to-severe asthma (NCT05104892). As mentioned earlier, BTK deficiency is characterized by decreased B cell level and serum immunoglobulin level. It is expected that patients with BTK deficiency be protected from atopy such as allergic rhinitis, asthma, and atopic dermatitis (eczema). Surprisingly, in a case report, a 7-year-old boy with agammaglobulinemia presented with allergic rhinitis, severe papular urticaria, asthma symptoms, and a positive skin prick test to aeroallergens and food allergens. He had a mutation in the *BTK* gene revealed by genetic analysis [114]. The administered BTKIs in asthma are shown in Table 7.

## BTKIs adverse events

Both on-target and variable off-target activities of BTKI on the cellular process are suggested to be linked with adverse events (AEs), since some AEs cannot be explained by BTK inhibition alone [115]. Clinically, AEs develop during long-time therapy with BTKIs because of unlimited inhibition of BTK, and consequently lead to significant rates of dosage reduction or treatment cessation. So, toxicity and AEs profile of BTKIs are related to their pattern of kinase binding [116]. The most observed AEs of BTKIs include bleeding, rash, diarrhea, and atrial fibrillation (AF).

Bleeding is assumed to be related to the effect of BTKI on BTK and TEC family proteins and their role in collagen-induced platelet aggregation, GPIb-IX, and integrin αIIbβ3 [117–119]. Rash and diarrhea are epidermal growth factor receptor (EGFR)-related AEs in BTKI-treated patients. AF is attributed to the BTKIs effect on C-terminal Src kinase (CSK) [120]. Another suggested mechanism of BTKIs-related AF is the inhibition of PI3K signaling which is responsible for cardiac protection under stress and is regulated by BTK and TEC family proteins [121]. Clinicians are advised to monitor cardiac symptoms, such as light-headedness, syncope, and palpitations, in patients on all BTKIs [116]. All BTKi AEs are shown in Table 8.

**Table 8:** Adverse events of BTK inhibitors

BTK inhibitor	Significant trial/study	Immune-mediated disease	Adverse event
***Ibrutinib***	Phase III	Patients with relapsed/refractory (R/R) CLL	Diarrhea, Fatigue, Nausea, Pyrexia, Anemia, Neutropenia, Thrombocytopenia, Pneumonia, AF, A Subdural Hematoma in 1 patient [122]
	Phase III	Patients with CLL/SLL	Cough, Hypertension, Diarrhea, Fatigue, Nausea, Pyrexia, Anemia, Neutropenia, Thrombocytopenia, Pneumonia, AF [123, 124]
	Case reports	Patient with CLL	Lymphocytosis (an early AE), Migratory Arthralgias and Fatigue (a late AE), Myalgia arthralgia, Migratory Arthralgias, Ventricular Arrhythmias, Reduced QT Duration, Cardiac Death [116, 125–127]
	Phase III	Patient with CLL	Minor Bleeding (Low-Grade Ecchymoses And Petechiae) with Impaired Platelet Function and Decreased Platelet Count rather than Thrombocytopenia, Major Bleeding (Less Frequently), Bleeding In The Presence or Absence Of Thrombocytopenia, Invasive Fungal Infections, such As Pneumocystis Jirovecii (also in XLA patients) and Aspergillus Fumigatus [122, 123, 128–139, 143]
	Phase Ib/II	First-Line and Relapsed/Refractory CLL	Hypertension [145]
	Phase III	Patients with Lymphoid malignancy including CLL, MCL, WM	New-Onset or Worsened High Blood Pressure accompanied by Arrhythmia, Myocardial Infarction, Heart Failure, Stroke, and Cardiovascular Death [146]
	Case reports	Patients with CLL	Non-Palpable Asymptomatic Petechial Rash, Pruritic Palpable Rash, Erythema Nodosum, Brittle Fingernails or Toenails, And Formation of Vertical Nail Ridges [147–149]
***Acalabrutinib***	Phase III	Patients with CLL	Headache, Diarrhea, Fatigue, Cough, Upper Respiratory Tract Infection, Arthralgia, Bleeding Events Such As Contusion And Petechiae, Neutropenia, Anemia, Thrombocytopenia, Urinary Tract Infection, Pneumonia, Dyspnea, Back Pain, AF, Acute Myocardial Infarction, Brain Injury, And Cardiac Failure [157]
	Phase III	Patients with relapsed or refractory CLL	Increased Levels Of Alanine Aminotransferase, Hepatotoxicity, And Major Bleeding [159]
	Phase II	Patients with relapsed/refractory CLL and treatment-naive CLL	Increased Levels Of Alanine Aminotransferase, Hepatotoxicity, And Major Bleeding, Sepsis [158, 160]
***Zanubrutinib***	Phase II	Patients with R/R CLL/SLL	Neutropenia, Thrombocytopenia, Lung Infection/Pneumonia, Upper Respiratory Infection, And Anemia [165]
	Phase III	Patients with del(17p) CLL/SLL	Contusion, Diarrhea, Nausea, Constipation, Rash, Back Pain, Cough, Arthralgia, Fatigue, Minor and Major Bleeding, Bruising, Dermatological Malignancies, Non-Skin Second Malignancies, AF, Sepsis Secondary To Pseudomonas, Melanoma, Acute Renal Failure, And *4 of the patients died in this trial: 2 due to disease progression, 1 due to an adverse event after disease progression (acute kidney injury), 1 after disease progression due to septic shock [166]
	Phase III comparing zanubrutinib versus ibrutinib	Patients with WM	Both AF and hypertension in lower rates for zanubrutinib than ibrutinib, Diarrhea in lower rates for zanubrutinib than ibrutinib [166, 168]
***Tirabrutinib***	Phase II	Naïve patients or patients with relapsed/refractory WM	Rash, Neutropenia, Lymphopenia, Leukopenia, Diarrhea [127, 169–171]
***Fenebrutinib***	Phase II	Patients with active rheumatoid arthritis	Upper Respiratory Tract Infections, Nausea, Headache, And Anemia [172]

### Ibrutinib

While evaluating the efficacy and safety of ibrutinib in phase III RESONATE (PCYC-1112) in patients with relapsed/refractory (R/R) CLL with a median age of 67 years, the most common AEs were diarrhea, fatigue, nausea, pyrexia, anemia, neutropenia, thrombocytopenia, pneumonia, and AF. A subdural hematoma was reported in 1 patient in this trial [122]. In phase III studies RESONATE-2 (PCYC-1115-CA) and ILLUMINATE in patients with CLL/SLL and with a median age of 73 years, the most common AEs were cough, hypertension, and AF, in addition to the AEs mentioned earlier [123, 124]. In a case report, a 68-year-old man with CLL received ibrutinib. His initial response was lymphocytosis. After 6 months, he reported migratory arthralgias and fatigue [116]. Myalgia and arthralgia, mostly migratory arthralgias, are observed in a retrospective analysis of CLL patients treated with ibrutinib [125]. With longer-term follow-up, some cases are reported with ventricular arrhythmias and cardiac death, as ibrutinib is associated with reduced QT duration [126, 127]. Another AE is minor bleeding (low-grade ecchymoses and petechiae) emerging in up to two-thirds of patients associated with impaired platelet function and decreased platelet count rather than thrombocytopenia [128]. Major bleeding is reported less frequently in 2–9% of patients [122, 123, 129]. Bleeding among ibrutinib-treated patients can occur in the presence or absence of thrombocytopenia [130–135]. Some opportunistic infections, especially invasive fungal infections, such as *Pneumocystis jirovecii* and *Aspergillus fumigatus* have emerged in patients with CLL on BTKIs, particularly on ibrutinib [136–139]. *Aspergillus fumigatus* induces BTK phosphorylation in macrophages, and impairs nuclear factor of activated T-cells (NFAT) and nuclear factor kappa-light-chain-enhancer of activated B cells (NF-κB) responses [140], ITK kinase [141], and M1 polarization in macrophages [142]. These mechanisms increase the susceptibility of ibrutinib-treated patients to fungal infections. There are a few reports of *Pneumocystis jirovecii* in XLA patients, as the ibrutinib-sensitive TEC kinase is a substitute for BTK in non-B-cells [143]. Thus, both inhibition of BTK and TEC may predispose XLA patients to fungal infection. Ibrutinib inhibits the platelet adhesion to lymphatic endothelial cells through phospho-SRC/spleen tyrosine kinase (SYK) and C-type lectin-like receptor 2 (CLEC-2) that is another proposed mechanism for increased rates of invasive fungal infections [144]. In phase Ib/II PCYC-1102 and extension study PCYC-1103 with up to 8 years of follow-up, the most sustained AE was hypertension in 28% of patients [145]. The proposed mechanism is the inhibition and downregulation of PI3K-p110α and nitrous oxide production [121, 146]. From 2009 to 2016, hypertension rates were studied in 562 patients treated with ibrutinib for malignancies, amongst which 440 (78.3%) patients developed new or worsened high blood pressure over a median follow-up of 30 months. The effect of new-onset or worsened hypertension on major cardiovascular events was assessed; hypertension was accompanied by arrhythmia, myocardial infarction, heart failure, stroke, and cardiovascular death [146]. Non-palpable asymptomatic petechial rash (which is associated with ibrutinib-induced platelet dysfunction), pruritic palpable rash (which is associated with EGFR inhibition and infiltration of the inflammatory cells) [147, 148], erythema nodosum, brittle fingernails or toenails, and formation of vertical nail ridges are observed in two-thirds of patients on ibrutinib [149]. Conversely, another mechanism underlying rash is the ibrutinib-induced increase of EGFR expression in dermal fibroblasts in the HDF3CGF system [150–152]. Unfortunately, 60% of patients on ibrutinib in the long-term had acquired resistance to covalent inhibitors, caused by cysteine C481 to serine substitution in BTK [153–155]. Two possible explanation for BTKI’s contribution to the occurrence of AF is discussed earlier; another possible explanation for ibrutinib-induced AF is the simultaneous binding to HER2 and HER4, whereas acalabrutinib inhibits HER4 and TEC, but not HER2; zanubrutinib inhibits TEC and HER4, but not HER2; tirabrutinib inhibits TEC but neither HER4 nor HER2. Ibrutinib’s simultaneous targeting of HER2 and HER4 is suggested to be responsible for AF [156].

### Acalabrutinib

Efficacy and safety of acalabrutinib was evaluated in the phase III study ELEVATE-TN in patients with CLL with a median age of 70 years. The most common AEs included headache, diarrhea, fatigue, cough, upper respiratory tract infection, arthralgia, bleeding events such as contusion and petechiae, neutropenia, anemia, thrombocytopenia, urinary tract infection, pneumonia, dyspnea, back pain, AF, acute myocardial infarction, brain injury, and cardiac failure [157]. Headache is uniquely observed with acalabrutinib; nearly 70% of patients experienced headaches during weeks 1 to 3 of treatment [158]. In phase III ASCEND in patients with relapsed or refractory CLL with a median age of 67 years, increased levels of alanine aminotransferase, hepatotoxicity, and major bleeding were also reported in addition to the earlier mentioned AEs [159]. The same AEs in addition to sepsis were reported in the phase II ACE-CL-001 [158, 160]. In clinical studies, rates of discontinuation due to AEs are lower with acalabrutinib rather than ibrutinib (9–11% at 28.3-month follow-up) [157]. In in vitro study of human platelets, acalabrutinib does not inhibit TEC, suggesting a reduced number of bleeding cases [161, 162]. Acalabrutinib has a lower rate of AF in comparison with ibrutinib [158]. As mentioned earlier, diarrhea is the most common AE in patients on BTKIs, which occurs before month 6 of treatment [122, 157, 163], and the rates of diarrhea reported in patients on acalabrutinib are similar to those on ibrutinib [158, 164].

### Zanubrutinib

In a phase II study, zanubrutinib was evaluated in Chinese patients with R/R CLL/SLL with a median age of 61 years; the most common AEs were neutropenia, thrombocytopenia, lung infection/pneumonia, upper respiratory infection, and anemia [165]. In the phase III SEQUOIA trial on patients with del(17p) CLL/SLL with a median age of 70 years, the most common AEs were contusion, diarrhea, nausea, constipation, rash, back pain, cough, arthralgia, fatigue, minor bleeding, bruising, dermatological malignancies, non-skin second malignancies, AF, sepsis secondary to pseudomonas, melanoma, and acute renal failure, and 4 of the patients died in this trial; two due to disease progression, one due to an adverse event after disease progression (acute kidney injury), and one after disease progression due to septic shock [166]. In another study, 0.3–2.2% of major bleedings were seen in zanubrutinib-treated patients [167]. Based on the results from clinical trials, fewer AF cases were reported in patients on zanubrutinib or acalabrutinib (mentioned earlier) than ibrutinib. Moreover, in the phase III trial ASPEN study, zanubrutinib versus ibrutinib in patients with WM was compared. Both AF and hypertension were reported in lower rates for zanubrutinib than ibrutinib with a median follow-up of 19.4 months [168]. Thus, treatment with zanubrutinib or acalabrutinib leads to fewer AF cases [156]. Among ibrutinib-treated patients, the frequency of diarrhea is reported in 32% of patients, while it is reported in 21% of patients treated with zanubrutinib, which is associated with a less potent inhibition of EGFR [166].

### Tirabrutinib

In a low-patient-enrolled phase II study in treatment-naïve patients or patients with relapsed/refractory WM (27 patients in total), the most common AEs were rash, neutropenia, lymphopenia, and leukopenia. The trial was a short-term follow-up and the available dataset on AEs was limited, but the trial met the primary endpoint [169]. In clinical trials, diarrhea was reported in 7–44% of patients receiving tirabrutinib [127, 170, 171].

### Fenebrutinib

In the phase II ANDES study in patients with active RA who were on fenebrutinib, the most common AEs were upper respiratory tract infections, nausea, headache, and anemia [172].

## Conclusion and future directions

BTKI target BCR signaling cascades that are responsible for both normal and malignant B cells’ survival and proliferation. BTKI binds to the ATP-binding site of BTK and blocks the phosphorylation of kinases in the BCR signaling cascade and also reduces B cell mitochondrial respiration resulting in less B cell activation, less secretion of b cell-derived pro-inflammatory cytokines, and less co-activation of T cell. First, second, and third-generation, reversible, and irreversible BTKIs, based on binding mode, are all developed and evaluated in clinical trials. Clearly, ibrutinib as a first-generation BTKI and the best-studied BTKI so far has already shown remarkable efficacy in the treatment of various B cell malignancies such as high-risk CLL, MZL, WM, relapsed/refractory MCL, and chronic GvHD. Ongoing clinical trials have clarified that BTKIs, particularly highly selective second and third-generation BTKIs, can provide therapeutic options in immune-mediated diseases where B cells and T cells are responsible for the disease etiopathogenesis. Application of BTKIs is still challenging due to the diverse AEs and as it cannot guarantee adequate safety and efficacy in immune-mediated diseases. Therefore, further research on the unexplored aspects of BTKI are strongly recommended.

## Data Availability

Not applicable.
